# Mapping and exploring health systems’ response to intimate partner violence in Spain

**DOI:** 10.1186/1471-2458-13-1162

**Published:** 2013-12-10

**Authors:** Isabel Goicolea, Erica Briones-Vozmediano, Ann Öhman, Kerstin Edin, Fauhn Minvielle, Carmen Vives-Cases

**Affiliations:** 1 Epidemiology and Global Health Unit, Department of Public Health and Clinical Medicine, Umeå University, Umeå, Sweden; 2 Public Health Research Group, Department of Community Nursing, Alicante, Spain; 3 Preventive Medicine and Public Health and History of Science, Alicante University, Alicante, Spain; 4 Department of Nursing, Umeå University, Umeå, Sweden; 5 CIBER of Epidemiology and Public Health (CIBERESP), Barcelona, Spain

**Keywords:** Health system, Health policy, Intimate partner violence, Spain, Mixed methods, Content analysis

## Abstract

**Background:**

For a comprehensive health sector response to intimate partner violence (IPV), interventions should target individual and health facility levels, along with the broader health systems level which includes issues of governance, financing, planning, service delivery, monitoring and evaluation, and demand generation. This study aims to map and explore the integration of IPV response in the Spanish national health system.

**Methods:**

Information was collected on five key areas based on WHO recommendations: policy environment, protocols, training, monitoring and prevention. A systematic review of public documents was conducted to assess 39 indicators in each of Spain’s 17 regional health systems. In addition, we performed qualitative content analysis of 26 individual interviews with key informants responsible for coordinating the health sector response to IPV in Spain.

**Results:**

In 88% of the 17 autonomous regions, the laws concerning IPV included the health sector response, but the integration of IPV in regional health plans was just 41%. Despite the existence of a supportive national structure, responding to IPV still relies strongly on the will of health professionals. All seventeen regions had published comprehensive protocols to guide the health sector response to IPV, but participants recognized that responding to IPV was more complex than merely following the steps of a protocol. Published training plans existed in 43% of the regional health systems, but none had institutionalized IPV training in medical and nursing schools. Only 12% of regional health systems collected information on the quality of the IPV response, and there are many limitations to collecting information on IPV within health services, for example underreporting, fears about confidentiality, and underuse of data for monitoring purposes. Finally, preventive activities that were considered essential were not institutionalized anywhere.

**Conclusions:**

Within the Spanish health system, differences exist in terms of achievements both between regions and between the areas assessed. Progress towards integration of IPV has been notable at the level of policy, less outstanding regarding health service delivery, and very limited in terms of preventive actions.

## Background

Men’s intimate partner violence (IPV) against women, defined as “*any behaviour within an intimate relationship that causes physical, sexual or psychological harm, including acts of physical aggression, sexual coercion, psychological abuse and controlling behaviours*”, is widespread [1,2]. The most recent global estimates of violence against women show that 35% of women worldwide have experienced physical and/or sexual intimate partner violence or non-partner sexual violence [3]. Within the EU-27, between 20% and 25% of all women have experienced IPV at least once in their lifetime [4].

IPV has devastating effects on the health and wellbeing of women and children [1,3,5,6]. Health services can play a key role in the prevention and management of IPV because of the many harmful effects on health they must attend to, and also due to the fact that women may access health services more often than other public services. Health care, and especially primary health care, can be an IPV survivor’s first and only point of contact with public service professionals [7,8]. Moreover, this contact can open doors for improved health and wellbeing; research shows that trained health providers improve IPV detection and referral to specialist violence agencies [9] - where intensive advocacy interventions can be provided [10]. A recent randomised controlled trial conducted in Australia showed that screening and brief counselling in primary care settings improved doctors’ follow up inquiry about women’s and children’s safety at 12 months, but did not improve other outcomes, such as quality of life, safety behaviour or anxiety [11].

There is general consensus that the health sector should carry out the following actions [1,6,8,12,13]: ask all women about violence, stay alert to possible signs and symptoms, provide health care assistance and register all cases, provide information on available resources, coordinate with other professionals and institutions, and provide evidence of the magnitude and seriousness of IPV. All these actions should be carried out while ensuring privacy and confidentiality, in a supportive environment where women’s experiences are validated and their decisions are respected [1]. However, integration of these actions varies significantly between countries, regions, and even between health care facilities [12,14]. There have been several studies that assess how health providers and/or health facilities respond to IPV, in terms of exploring knowledge, opinions and practices; measuring possible changes in connection with interventions; and focusing specifically on adopting IPV screening [9,15-23]. However, there is less research that explores the response at the health system level [8,13]. It is important to fill this gap, since successful and sustained policy integration in the health sector cannot be achieved through isolated strategies directed towards individuals and/or health facilities alone, rather they should target larger health system functions, including: i) governance, ii) financing, iii) planning, iv) service delivery, and v) monitoring and evaluation [24,25]. Research shows that in order to sustain long-term improvements in the health sector response to IPV, changes should be made not only at the individual provider/facility level through training, but should also involve changes in health policies, protocols, managerial structures and practices [13,26,27].

This study aims to map and explore the integration of the IPV response in the Spanish national health system. In Spain the “Gender Based Violence Law”, enacted in 2004, has been recognized as one of the most progressive and comprehensive pieces of legislation on gender-based violence worldwide. The law specifically addresses the responsibilities of the health sector [28-31]. The Law establishes an array of measures, including judicial system reforms, and the implementation of a comprehensive network of social services aimed at protecting the rights and security of women exposed to IPV. The Law also establishes the need to implement preventive measures to challenge gender inequality at the broader social level. Regarding the role of the health sector, the Law states that health services should be aware of possible cases of violence, manage them, and engage in a multidisciplinary response in coordination with other institutions and sectors. In order to monitor these actions, the ‘National Commission against Gender Based Violence’ (NCAGBV) was created within the Inter-territorial Council of the National Health System, which is the highest level of decision-making within the Spanish health system [32]. The NCAGBV is comprised of delegates from each autonomous region and national representatives of the Ministry of Health.

By describing the situation in Spain and highlighting its strengths and challenges, we aim to provide information useful not only for this country, but for informing health systems in general in their efforts towards achieving IPV integration.

## Methods

### The setting: IPV and the Spanish health system

Though Spanish legislation refers to gender-based violence, the concept used in this study is IPV. During data collection it became clear that the health sector response has focused specifically on IPV, and less so on other forms of gender based violence–i.e. sexual assault by non-partners, trafficking, female genital mutilation-that have just recently begun to be addressed. According to a survey conducted with 11,000 women using primary health care facilities in Spain, the reported lifetime prevalence of IPV in 2007 was 32% [33].

The Spanish health system is highly decentralized. The 17 autonomous regions–each with its own parliament and government-and 2 autonomous cities located in the North of Morocco are in charge of health planning, public health, and management of health services. Health services are offered through a network of primary health care centres, which is made up of a multidisciplinary team of family doctors, nurses, social workers, midwives and paediatricians, and hospitals. In some regional health systems there are also other specialized services offered at the community level, which coordinate closely with primary health care facilities but are not part of them. These include mental health, reproductive health, and addictive behaviour units.

**Figure 1:**
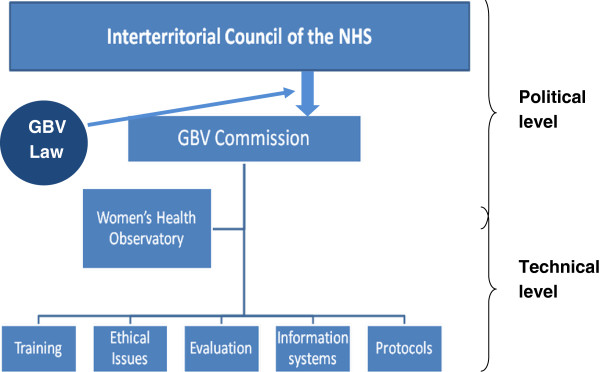
Bodies created within the Spanish national health system to coordinate and monitor IPV response, grounded on the 2004 GBV Law.

At the level of regional health systems (RHSs), coordination for IPV is the responsibility of regional delegates to the NCAGBV and civil servants. These civil servants, together with representatives from academic institutions and other government agencies with expertise or responsibilities related to IPV, participate in 5 working groups that have been created by the NCAGBV to coordinate actions related to: 1) training, 2) evaluation, 3) protocols, 4) information systems and indicators, and 5) ethical issues. The Observatory of Women’s Health, a technical body created within the Spanish Ministry of Health, acts as secretariat of the NCAGBV and gives support to the working groups [32,34-38]. See Figure 1 for a summary of the different bodies created in the Spanish health system to promote and monitor the response to IPV (Figure 1). In Spain, the integration of IPV response has focused on first-line health services, i.e. primary health care centres. Progressively, other specialized services -such as mental health clinics, hospital emergency departments and other specialized departments-are beginning to be incorporated.

In Spain the primary responsibility for health system implementation lies at the regional level, therefore, in this study we explored the 17 regional health systems of the autonomous regions; the autonomous cities of Ceuta and Melilla, located in the North of Morocco were excluded since their health systems depend on a different structure (INGESA).

### Research methodology

**Figure 2:**
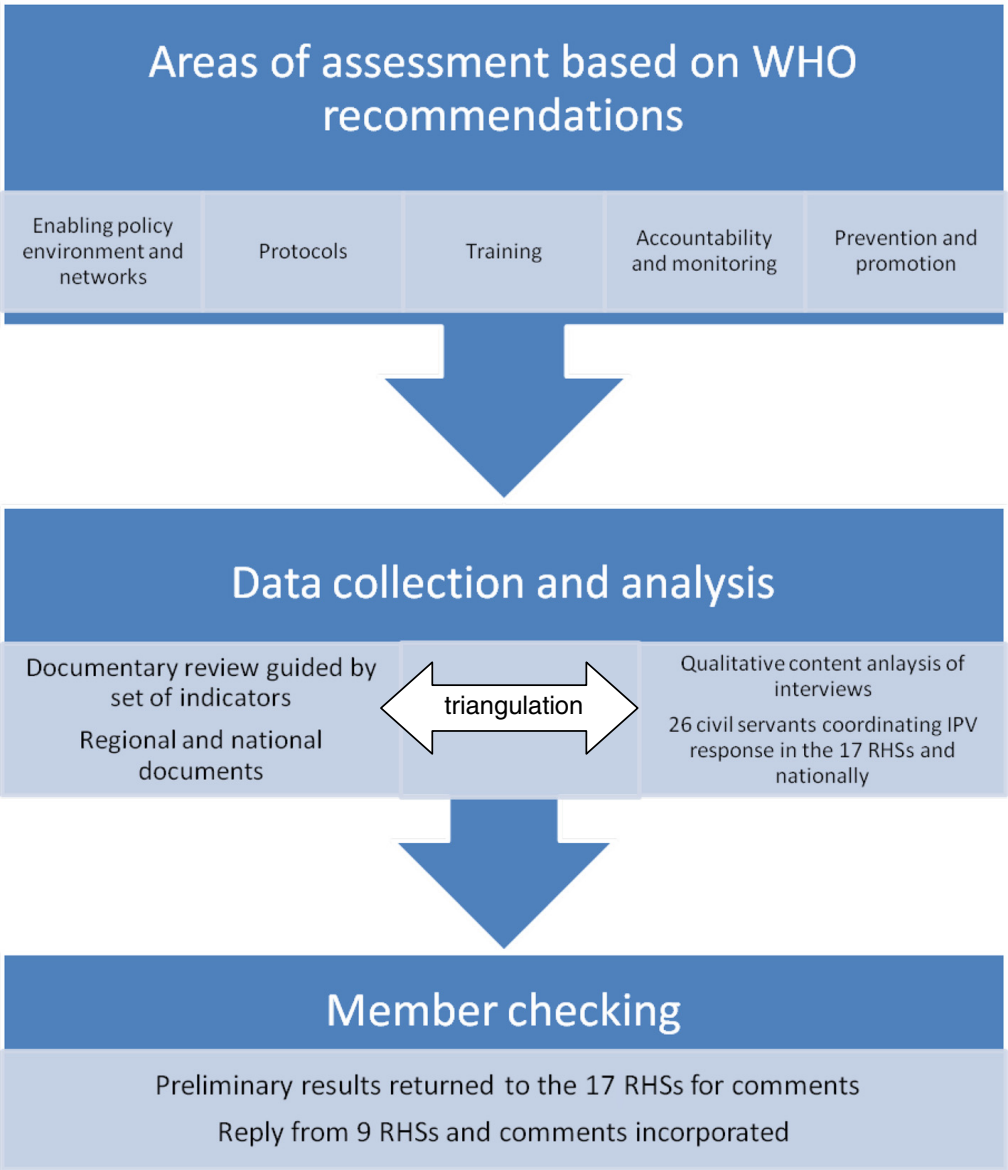
Methods for data collection and analysis.

This study aims to map and explore the integration of IPV response in the Spanish national health system. We conducted a systematic review of public documents regarding the health system’s response to IPV in Spain as well as qualitative interviews with key informants within the Spanish health system. Based on the WHO recommendations for the health sector response to violence against women [1,6], five key areas of assessment were identified: 1) policy environment and networks, 2) protocols and guidelines to direct the healthcare response, 3) training of health professionals, 4) accountability and monitoring mechanisms, and 5) prevention and promotion. For each of these areas, quantitative and qualitative information was collected. Information collected through the documentary review was used to map the integration of IPV within Spain’s decentralized health systems, while qualitative information from the interviews permitted a deeper exploration of the process. For a summary of the methodological steps, see Figure 2. A more detailed description of the methodology can be found elsewhere [39].

#### Mapping: systematic review of public documents

Content analysis was conducted as described by Ortiz-Barreda and Vives Cases [28-30]. Existing documents were systematically analyzed to assess 39 indicators-from the five areas described above-in each of the 17 RHSs. These indicators were selected based on WHO and national guidelines. However, during data collection some indicators that were considered important were not available, i.e. even if indicators related to funding for IPV programmes would have been important to collect, they were unavailable. Regional documents reviewed included laws, health plans and protocols concerning the issue of IPV within the autonomous health systems. National documents reviewed included reports of IPV for the years 2005-2011 (see Additional file 1 for a summary of the main documents reviewed). For each RHS, indicators were assessed as present or absent.

#### Exploring: qualitative interviews with key informants

Individual interviews were conducted from July 2012 to March 2013, with a theoretical sample of 23 key informants from the autonomous regions and three informants at the national level. Informants in the autonomous regions were civil servants of the RHSs in charge of coordinating the health-sector response to IPV. Their backgrounds varied; the majority were medical doctors (14), although there were also nurses (3), psychologists (2), one anthropologist, one midwife, one social worker, and one sociologist. They were all participating–or had participated-in the working groups and some of them had also participated in the NCAGBV. One informant per RHS was contacted first. In some RHSs another informant was included due to his/her experience in certain areas of interest to the study. Informants at the national level were representatives of the Observatory of Women’s Health and academic institutions–one had a pharmaceutical degree and was in charge of the Observatory of Women’s Health, another was a nurse working at the Observatory, and the third was a midwife working in an academic institution who also held an advisory role for the Women’s Health Observatory. We selected civil servants at the managerial level, and not politicians, because they remain in their positions for a longer time and play a more direct and active role in implementing the health system’s response to IPV in their regions. They were chosen based on their status as privileged informants-able to contribute significantly to our research-through theoretical sampling. All of the prospective informants who were chosen agreed to participate. Fifteen of the interviews were conducted face to face, 11 were phone interviews, and the average duration was one hour. All but two of the participants were women. First contacts were facilitated through the National Observatory of Women’s Health and subsequent contacts came from interviewees themselves, through snowball sampling. The average duration of the interviews was one hour; 16 interviews were conducted face to face, while 10 were phone interviews. The interviews started with an open question encouraging participants to describe how the process of integrating IPV has occurred in their region–or nationally in the case of national level informants. Afterwards, questions were asked in order to explore the five areas of interest.

All the interviews were held in Spanish, recorded and transcribed verbatim. Transcripts were imported into the software Atlas.ti-5 to manage the analytical process. We used qualitative content analysis as described by Graneheim and Lundman [40], focusing on the manifest content of the text. First, we identified the meaning units that referred to the five major content areas previously described. Within each of the major content areas, identified meaning units were condensed and later coded. Afterwards, codes were grouped together to build categories. The coding and analysis was done using the original Spanish.

Data collected through the individual interviews served to triangulate and to complement the information previously gathered through the documentary review, while information from the documentary review served to further explore regional particularities during the qualitative interviews. Preliminary results were sent to the participants for member checking: nine of them responded with comments that were incorporated into the final versions of the tables. Additional file 2 summarizes the application of the RATS guidelines for qualitative research, to assess this manuscript.

The study was approved by the Ethical Committee of the University of Alicante. Each participant in the study was asked to provide written informed consent prior to conducting the interviews. Information that could identify the respondents was eliminated.

## Results

**Figure 3:**
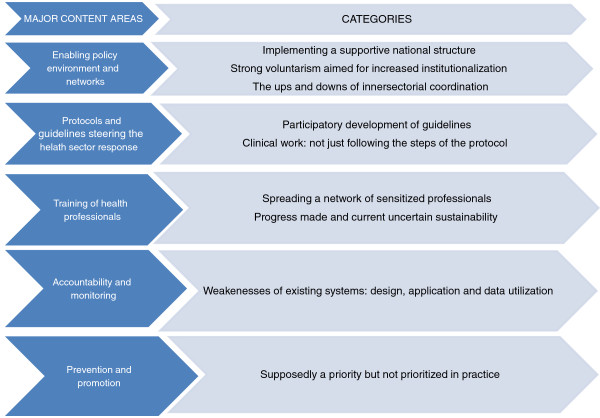
Summary of major content areas explored and emerging categories.

Results are presented for each of the five areas assessed; the results from the documentary review are presented in a table, which is followed by the findings from the analysis of the qualitative interviews. Figure 3 presents the summary of the five major content areas, and the categories emerging from the qualitative content analysis of the interviews.

### Policy environment and networks

Fifteen out of 17 of the Regional Health Systems had passed Autonomic Laws against IPV that explicitly mention the health sector’s responsibilities. However, the inclusion of IPV within regional health plans occurred in only 7 out of 17 RHSs, and the integration of IPV indicators within “program contracts”-agreements between the managerial and the operational levels of the health system that prioritize certain health indicators to be achieved-occurred only in 7 out of 17 RHSs. In 13 RHSs there were informal teams in charge of coordinating IPV actions, but only 6 RHSs had a person or team officially designated. Thirteen out of 17 autonomous regions had intersectorial committees, and 15 had developed protocols for an intersectorial response to IPV that included the health sector. See Table 1.

**Table 1:** Indicators related to policy environment and networks (Published documents and committees as per December 2012)

		**TOTAL n (%)**^**5**^	**Andalucía**	**Aragon**	**Asturias**	**Baleares**	**Canarias**	**Cantabria**	**C-La Mancha**	**C-Leon**	**Cataluña**	**C Valenciana**	**Extremadura**	**Galicia**	**La Rioja**	**Madrid**	**Murcia**	**Navarra**	**País Vasco**
**Criteria**	**Indicator**																		
Policies and procedures in place in health system	Autonomic Law against IPV mentions explicitly health sector response	15 (88)	+	+	+	+	+	+	-	+	+	-	+	+	+	+	+	+	+
	Latest autonomic health policy/plan includes IPV as health problem	7 (41)	+	-	+	+	-	+	-	+	-	-	-	-	+	+	-	-	-
	IPV management included in primary health care portfolio	12 (71)	+	+	+	-	+	+	-	+	+	+	+	+	+	+	-	-	-
	IPV indicators included in primary health care program contracts^1^	7 (44)	+	-	+	-	-	+	-	+	-	+	NA	+	-	+	-	-	-
Engagement at the managerial level	Team of people who work together coordinating IPV activities within the health system (official or not but functioning)^2^	13 (76)	+	+	+	-	-	-	+	+	-	+	+	+	+	+	+	+	+
	Exists a person or group officially recognized for managing the health system’s response to IPV^3^	6 (35)	-	-	+	-	-	-	-	-	-	-	+	+	-	+	-	+	+
Health sector integrated in an intersectorial response	Protocol for intersectorial response to IPV published and includes health sector^4^	13 (76)	+	+	+	+	+	-	+	+	+	+	+	-	+	-	-	+	+
	Exists an intersectorial body for dealing with IPV (committee, plan, etc.) in which health sector included	15 (88)	+	+	+	+	+	+	+	+	+	+	-	-	+	+	+	+	+

^1^In certain autonomous regions, like C Valenciana and La Rioja, health system’s management is not based on “program contracts”.

^2^A team existed in Canarias until 2010, but not longer afterwards. At team existed in Baleares until November 2011.

^3^There was somebody designated in Cataluña RHS but no longer.

^4^In Murcia the protocol was developed before December 2012, but was passed in 2013. In Madrid there are plans at the municipal level, but not at the regional level.

^5^Total refers to the number of RHSs in which the indicator was present, against the total number of RHSs. The raw number and the percentage (in brackets) are provided.

#### Implementing a supportive national structure

Participants acknowledged that the 2004 Gender Based Violence Law constituted a cornerstone for building an enabling policy environment. The law detailed the health sector’s responsibilities and supported earlier regional initiatives, to guide the main lines of work on IPV in the national health system. It also pushed for the development of enabling structures within the national health system, such as the NCGBV and the working groups. These structures enhanced cohesion between the RHSs and made it possible to reach consensus regarding guidelines, indicators, and training objectives. They also served to build an inter-regional network, where RHSs have been able to exchange experiences and good practices and support each other’s efforts. Worth highlighting is that while the NCGBV was comprised of policy makers, the working groups were constituted by a variety of professionals, both civil servants in the regional health systems and professionals involved in clinical work. The guiding role of the Observatory was highly valued by participants.

*Within this space you get working guidelines, funding, coordination is established, and it’s a cornerstone. It’s a meeting point, and the fact that we* [the RHSs] *have to submit an annual report puts everybody to work, it’s a strategy that develops cohesion. I think that the Observatory fulfils that function. E6*

#### Strong voluntarism aimed at increased institutionalization

Participants expressed the importance of building teams of people interested in IPV to coordinate the activities in each of the RHSs. Those teams of civil servants with expertise on IPV had close links with clinical practice and had strong motivations to mobilize the work on IPV within the RHSs. In some regions, informal working teams-that included both civil servants at the managerial level and professionals working at health care facilities-were created in order to better accommodate the needs of first line health care practitioners. However, the civil servants in charge of IPV within the RHSs had to overcome three main barriers: 1) they had other responsibilities besides IPV, and many lacked official designation, making them vulnerable to political turnovers; and 2) the lack of commitment of certain political stakeholders. In general, these stakeholders had a medicalised approach to IPV and consequently might not necessarily consider investing in actions aimed at prioritizing IPV and improving the response of health services. This second barrier was described by one of the interviewees:

*When I started working in 2006, since there was money for IPV I went to see my boss and said: “Hey, you should give me some money to train on gender based violence”, and he asked me: How many women died in this autonomous community due to gender based violence last year? I said, “None”, and he continued: “Every day I have 10 deaths due to cardiovascular diseases, so you can understand I am going to allocate very little money to gender based violence”.E3*

Achievements in IPV response were considered to be a result of the motivation and voluntarism of specific individuals, whether policy makers, civil servants or clinicians. Voluntarism was highly valued, but at the same time participants recognized that it could not stand alone without institutionalization of the actions and structures that have been built.

#### The ups and downs of inter-sector coordination

Participants acknowledged that the health sector alone could not respond effectively to IPV and valued the coordinating efforts developed in the RHSs. They valued the existence of structures for such coordination-like commissions, agreements and protocols-but also acknowledged the key role of interacting face-to-face with those responsible in other sectors. Collaboration with other sectors was considered a facilitator for the establishment of referral networks between health care facilities and other services, in order to offer a comprehensive response to women exposed to IPV.

Coordinating between different sectors also brought challenges: 1) rivalry in terms of who should lead the process, 2) difficulties dealing with a weakened referral network due to cuts in social services, and 3) reaching agreement between different approaches. Regarding the latter, participants were especially worried about the conflict between a judicial approach to IPV-that focused on reporting-and a broader approach–favoured by health providers-that did not prioritize legal solutions.

Currently there is a tendency towards judicialisation that focuses on “report, report”. The law forces us to report, and women also have to report, in order to have the right to certain social benefits; but the path is a bit too rigid […]. The relationship with the judicial system is difficult, because it’s a very hierarchical system and very hermetic…, probably like medicine, but they are a State power, and that puts them at another level. E18

Although some concrete experiences of coordination between the educational and the health sectors were mentioned, participants considered that the former has generally been absent in these regional intersectorial coordination bodies.

### Protocols and guidelines steering the healthcare response

All of the 17 RHSs have published protocols/guidelines to guide health services’ response to IPV. Focus has been put on primary health care. The RHSs’ protocols fulfilled most of the WHO criteria that refers to health providers’ practices and emotional support. Regarding non-negotiable issues, two criteria were not explicitly mentioned in the majority of protocols: 1) that providers should not contact a woman’s partner (mentioned in only 8 out of 17), and 2) that providers should not refer women to traditional couples counselling (9 out of 17). The importance of ensuring confidentiality was addressed in 15 of the protocols, but only 4 explicitly mentioned the importance of keeping clinical records confidential. Only 3 of the RHSs incorporated routine inquiry for IPV into antenatal care. The need to explore the situation of children of victims of IPV, and the need to consider women in situations of vulnerability, appeared in 10 and 7 protocols respectively. See Table 2.

**Table 2:** Indicators related to protocols and guidelines (based on the latest published)

		**TOTAL n (%)**^**3**^	**Andalucía**	**Aragon**	**Asturias**	**Baleares**	**Canarias**	**Cantabria**	**C-La Mancha**	**C-Leon**	**Cataluña**	**C Valencia**	**Extremadura**	**Galicia**	**La Rioja**	**Madrid**	**Murcia**	**Navarra**	**País Vasco**
**Criteria**	**Indicator**																		
Clinical guidelines in place and implementation monitored^1^	Regional protocol and/or guidelines published	17 (100)	+	+	+	+	+	+	+	+	+	+	+	+	+	+	+	+	+
Health providers’ practices. Protocol clearly includes regarding Primary health care:	The need to document what the woman says and collect forensic evidence if needed	16 (94)	+	+	+	+	+	+	+	+	+	+	-	+	+	+	+	+	+
	The need to give information about crisis services and long-term services	16 (94)	+	+	+	+	+	+	+	+	+	+	-	+	+	+	+	+	+
	The need for safety planning	15 (88)	+	+	+	+	+	+	+	+	+	+	+	+	+	+	+	-	-
	The need for organize referrals (within the health care facility or external)	17 (100)	+	+	+	+	+	+	+	+	+	+	-	+	+	+	+	+	+
Emotional and psychosocial support. Protocol includes regarding Primary health care:	The need to validate women’s experiences	15 (88)	+	+	+	+	+	+	+	+	+	+	-	+	+	+	+	+	-
	The need to have non-judgmental attitudes	15 (88)	+	+	+	+	+	+	+	+	+	+	-	+	+	+	+	+	-
	The need to listen, assess the risk, evaluate the woman’s expectations and provide options	14 (82)	+	+	+	+	+	+	-	+	+	+	-	+	+	+	+	+	-
	The need to believe what the woman is saying, empathize and not belittle her experiences	15 (88)	+	+	+	+	+	+	+	+	+	+	-	+	+	+	+	+	-
Non-negotiable issues. Protocol includes regarding Primary health care that the health providers should:	Avoid contacting the woman’s partner^2^	8 (47)	-	-	-	-	+	+	-	-	+	+	-	-	-	+	+	+	+
	Avoid referring to traditional couple counselling^2^	9 (53)	+	+	-	-	+	-	-	+	-	-	-	+	+	+	+	+	-
	Ensure absolute confidentiality^2^	15 (88)	+	+	+	+	+	+	+	+	+	+	+	+	+	+	+	-	-
	Keep medical records somewhere confidential	4 (24)	-	-	-	-	-	-	-	+	-	+	-	-	-	+	+	-	-
	Ensure that woman’s decision prevail and she should be allowed to take action when she wants	13 (76)	+	-	+	+	+	+	-	+	+	+	-	+	+	+	+	+	-
Screening and clinical inquiry. Protocol includes regarding Primary health care:	Routine inquiry in antenatal care	3 (18)	-	-	-	-	+	+	-	+	-	-	-	-	-	-	-	-	-
	How to do appropriate clinical inquiry if signs	15 (88)	+	+	+	+	+	+	+	+	+	+	-	+	+	+	+	+	-
Link IPV with child protection	The protocol states the need to explore with women how their children are treated	10 (59)	+	+	-	+	+	+	-	+	+	-	-	+	+	+	-	-	-
Focus on women in situation of vulnerability	Protocol mentions the need to consider women in situations of vulnerability	7 (41)	+	-	+	+	+	-	-	+	-	-	-	-	+	+	-	-	-

^1^In some regions, like Castilla León there are more than one protocol, each addressing different aspects.

^2^In Aragón, even if the protocol does not explicitly include these aspects, they are addressed in the training. In La Rioja, even if it is not explicitly written to avoid contacting the partner, issues regarding difficulties when women came accompanied are addressed.

^3^Total refers to the number of RHSs in which the indicator was present, against the total number of RHSs. The raw number and the percentage (in brackets) are provided.

#### Participatory development of guidelines

Participants described the development of protocols as a participatory process, with a rich process of exchange between different levels. The national protocol for a health sector response to IPV, published in 2007, served as a base for the regions that had not published protocols up to that time, while the regional protocols that had been published before that date were also taken into account when developing the national protocol. Experiences from one autonomous region inspired the elaboration of protocols in other regions.

In order to develop our regional protocol, we first looked into the other protocols that had been published and their contents, and we developed our protocol based on that. I mean, we did not start from scratch, but since there were regions that were doing things, and they were doing them well, we took advantage of their experience. E7

At the regional level, participants expressed that the development of the guidelines was the result of team work, with the involvement of professionals from different sectors and levels of the RHSs. Civil servants at the managerial level participated, as did general practitioners, paediatricians, midwifes, social workers, gynaecologists, psychologists working in health care facilities, and actors from other sectors.

#### Clinical work: not just following the steps of the protocol

Participants considered that one of the main aims of the protocols was to guide and support clinicians’ actions in detecting and responding to cases of IPV. Protocols were perceived as facilitating clinicians’ work by detailing the actions they should carry out, and as one participant stressed:

The protocol is extraordinary since it leaves the professionals with no doubts. They know what to do at every moment, by following the protocol they know what to do, how to proceed, what to do on every occasion. The protocol leaves no room for improvisation. E15

However, as one participant pointed out *“when a protocol is developed, that’s not the end of the work, in fact the real work starts at that very moment, when professionals have to be engaged”* E19. Participants agreed that suspecting, detecting and questioning was not merely a matter of following the steps of a protocol but constitutes a learning process that professionals may or may not engage in. Dealing with IPV also demanded a different approach from providers, as the following quotation demonstrates:

The health professional doesn’t have all the answers, as when faced with biomedical problems; for example, faced with pneumonia, the health professional will know far more than the patient, […] if the patient follows the treatment, she/he will get better. With IPV, it’s not like this, […] the health professional lacks the answer in terms of what to do tomorrow, or the day after tomorrow, when facing her husband, her son […]. What she/he can do is open doors, give clues, and help the woman to make up her mind. E23

### Training of health professionals

Nine RHSs had training plans published, and 14 have a team of health providers with expertise on IPV able to engage in training others. These are mostly clinicians who were not dedicated full-time to this task but who could be available if needed. Measures to facilitate training included substitutions (in 5 of 17) and the inclusion of IPV training targets into “program contracts” (7 out of 17).

Eleven out of 17 RHSs have included issues of IPV into the training of doctor/nurse residents, but none of the autonomous regions have institutionalized training on GBV within undergraduate training. See Table 3.

**Table 3:** Indicators related to training of health professionals

		**TOTAL n (%)**^**4**^	**Andalucía**	**Aragon**	**Asturias**	**Baleares**	**Canarias**	**Cantabria**	**C-La Mancha**	**C-Leon**	**Cataluña**	**C Valenciana**	**Extremadura**	**Galicia**	**La Rioja**	**Madrid**	**Murcia**	**Navarra**	**País Vasco**
**Criteria**	**Indicator**																		
Training plan (as per 2011)	Official training plan published/institutionalized or formalized	9 (53)	+	-	+	+	-	+	+	+	-	+	-	-	-	+	+	-	
Trained professionals and training team (as per 2011)	Exists a group of trainers within the autonomous region	14 (82)	+	+	+	+	+	-	-	+	+	+	-	+	+	+	+	+	+
	Trainers with multidisciplinary profiles (three or more)- during 2011	16 (100)	+	+	+	+	+	+	+	+	NA	+	+	+	+	+	+	+	+
Measures to facilitate participation on training (as per 2011)	Substitutions^1^	5 (31)	-	-	-	+	-	+	-	-	+	+	NA	-	-	-	-	+	-
	Program contracts^2^	7 (47)	+	-	+	+	-	-	-	+	NA	+	NA	-	-	+	-	-	+
Supervision and reinforcement (as per 2011)	Training plan includes issues of supervision and support^3^	2 (14)	-	-	+	-	-	-	-	-	NA	-	-	NA	-	+	-	-	NA
Training included in undergraduate curricula (as per 2011)	Some training on GBV included in the curricula of health studies (undergraduate or specialization)	11 (69)	-	+	+	+	+	+	NA	+	-	+	-	+	+	-	+	+	NA
	GBV management officially included in the curricula of health studies	0 (0)	-	-	-	-	-	NA	-	-	-	-	-	-	-	-	-	-	NA

^1^In Baleares existed until 2011, but not in 2012 and beyond. In Asturias existed until 2010.

^2^In Madrid is explicitly included from 2012, before it was included as part of the “training on strategic lines”, being GBV included among these lines.

^3^In Aragón health providers receive actualized information on IPV; i.e. new policies.

^4^Total refers to the number of RHSs in which the indicator was present, against the total number of RHSs. The raw number and the percentage (in brackets) are provided.

#### Building a network of sensitized professionals

Participants considered that training activities organized in the RHSs served to build a network of health professionals who are sensitized and knowledgeable about IPV and who can support one another. Participation in courses on IPV have not been compulsory for health professionals, but a number of strategies to encourage and facilitate participation have been implemented, such as including training targets into “program contracts”, ensuring substitutions of professionals who attended training, and offering accreditation/certificates that could be used for career advancement. In some regions, training sessions included the participation of professionals from other sectors (police, judicial system, social services) in order to enhance collaboration and facilitate referrals from health professionals to other services.

Participants considered that training health professionals during their undergraduate studies is very important, but acknowledged that they have little power to influence universities’ curricula.

When I asked [the new medical and nursing residents] whether they had received training on IPV during their university education, none recalled having had such training […]; I mean, they have studied six years of medicine and nothing, no idea. E6

#### Progress made and current uncertain sustainability

Participants were convinced of the progress achieved. They recalled that they were pioneers when they started, facing opposition from providers at the clinical and managerial levels. They recalled that they were uncertain on how to proceed and lacked guidelines or expertise, but that they started because they felt there was a need to act on this problem.

There has been a specific training strategy [on IPV] directed towards health providers. We have been training for more than 10 years now, and that has been crucial […]. I notice a dramatic change; I mean, in the beginning when I talked about violence to health providers, they were resistant and replied that there was nothing that they could do: they were annoyed…, even those who voluntarily participated in IPV training workshops! E13

Evaluations have been scarce and were mainly limited to before and after assessment of knowledge or participants’ satisfaction with courses. Participants were uncertain regarding the extent to which training had made an impact on clinical practice. They also expressed that the lack of sustained strategies for supervising and supporting providers after they were trained might limit the impact of training sessions on changes in clinical practices.

They also considered that further training of more providers was still needed. In this sense, they were worried about the current economic situation. Funding for training activities–in general and specifically for IPV-had decreased, and strategies to facilitate participation in training had been dismantled. Additionally, providers’ salaries had decreased in some RHSs, and their workload had increased, resulting in a scenario in which professionals were not motivated to participate in training activities.

### Accountability and monitoring

As per 2011 indicators (the most recent available), four RHSs had collected and reported all of the 11 common indicators on IPV agreed upon in the NCGBV. Detection rates-defined as the number of new cases of violence among women age 14 and older detected by the health sector per 100,000 women of that age-varied widely between the RHSs. It ranged from 6.58 in Extremadura to 172.05 in Andalucía. The 11 common indicators did not provide information regarding the quality of the IPV services provided, but such indicators were collected in 2 RHSs, and another one had implemented reporting systems that will enable them to collect such information in 2013.

None of the RHSs had implemented measures for supporting the debriefing of professionals dealing with cases of IPV. Similarly, none had implemented systematic mechanisms to collect information on women’s experiences with the services, although studies had been conducted in some RHSs to assess women’s experiences with IPV and their perceptions of the services provided. See Table 4.

**Table 4:** Indicators related to accountability and monitoring

		**TOTAL n (%)**^**3**^	**Andalucía**	**Aragon**	**Asturias**	**Baleares**	**Canarias**	**Cantabria**	**C-La Mancha**	**C-Leon**	**Cataluña**	**C Valenciana**	**Extremadura**	**Galicia**	**La Rioja**	**Madrid**	**Murcia**	**Navarra**	**País Vasco**
**Criteria**	**Indicator**																		
Monitoring system that provide data on number of cases	All the 11 Common national indicators collected and reported in 2011 (in brackets number collected)	4 (25)	-	-	-	-	-	+	NA	+	-	-	+	-	+	-	-	-	-
	Increase on detection rates within health system from 2009 to 2011 (National Indicator 1)	6 (43)	-	+	+	-	-	+	-	+	NA	+	NA	NA	+	-	-	-	-
	Indicators regarding quality of services provided collected (13 to 15 or others similar) in 2011^1^	2 (12)	-	-	-	-	-	-	-	-	-	+	-	-	+	-	-	-	-
Debriefing support for health professionals	Procedures for debriefing support established	0 (0)	-	-	-	-	-	-	-	-	-	-	-	-	-	-	-	-	-
System to learn from women’s experiences of the service	Procedures to collect information from women’s experiences exist^2^	0 (0)	-	-	-	-	-		-	-	-	-	-	-	-	-	-	-	-

^1^In Castilla-Leon, the reporting system within the electronic clinical records allows the collection of such indicators, but since this has been recently implemented, those data were not available for use. In Aragón Indicators 13 and 15 are collected but not further used at the moment.

^2^In Madrid the Demographic survey is conducted annually since 2004 and collects data on service utilization, but not on experiences of women victims of IPV with existing health services.

^3^Total refers to the number of RHSs in which the indicator was present, against the total number of RHSs. The raw number and the percentage (in brackets) are provided.

#### Weaknesses of existing systems: design, application and data utilization

Participants mentioned several limitations of the existing reporting systems. First, the existing codes in most primary health care diagnostic tools were not specific for IPV. Using them may mean that cases of IPV might be split among diverse codifications or that certain codes might include not only IPV but also other forms of violence, hindering the collection of specific information.

When we started collecting the number of IPV diagnoses, we got very high numbers in comparison with other autonomous regions […]. We changed the diagnosis codes we were using, because we realized that what we were collecting was any type of violence, not just violence by a current or former partner. E10

Second, participants expressed that primary health care and specialized services often had different electronic systems that made it impossible to follow a case through different health care levels. This made follow-ups difficult and resulted in possibilities for duplication.

Third, applying the registration systems was more easily said than done. Participants considered that IPV registration was still in the early stages, with consequent errors and underreporting. Moreover, since cases of IPV were not detected frequently, professionals did not use IPV registration systems often, and when they had to do so, they were not necessarily familiar with them.

Fourth, data gathered was considered to be under-utilized. Besides reporting to the NCGBV, the information that emerged from collected indicators was not used for monitoring the work of the health services. Collected information was not returned to the health facilities that produced it, and participants considered that this could further discourage professionals’ proper registration of cases.

Finally, participants mentioned that the registration of IPV might generate conflicts/dilemmas among health providers. Despite the fact that access to electronic clinical records was limited to certain professionals, doubts about confidentiality and women’s safety were raised, for example, recorded IPV-related information could be seen by the woman’s partner on the computer screen during consultation. Writing down codes related to IPV-that might be seen by other health care professionals consulted by the woman-could further stereotype women, as one participant highlighted, “*Putting codes [regarding IPV] also means…, thinking twice, it means labelling, you keep on labelling women”. E12*

### Prevention and promotion–supposedly a priority but not prioritized in practice

It was not possible to collect quantitative information on this indicator, since none of the RHSs had institutionalized actions regarding primary prevention of IPV or the promotion of women’s empowerment within the health systems. Qualitative information portrayed primary prevention actions as important.

It is crucial to begin actions with young people, because if you start working at that moment, you can prevent violence before it happens: I think we need to focus on prevention, primary prevention, before IPV starts… Then I think we need to work with young people on prevention. E13

Qualitative interviews allowed the collection of information on specific preventive-promotional experiences in some of the RHSs, for example, coordinated work among women’s and community organizations, therapeutic work with groups of women addressing their *malaise*, and initiatives to promote more gender-equal and non-violent relationships among young people.

Participants stated that in some RHSs, actions aimed at prevention and/or promotion were starting to be incorporated into health plans and guidelines, but this was perceived as still incipient, not the focus of this first stage, and not generalized. They considered that engaging in prevention and promotion actions was very much dependent on the motivation and willingness of particular professionals and/or health care teams. The high demands that health care professionals already had with curative services were also mentioned as a further hindrance to the implementation of such activities.

[Regarding preventive activities]* No, no, we haven’t been able to engage in… It hasn’t been, it hasn’t surfaced as a priority, even if we knew it was a priority, but we had to focus on other issues […] we have tried to develop a guide for professionals on prevention; we are working on that but it is a very slow process, very slow […]. We have to try preventive actions and not merely be dependent on the willingness of certain individuals, because we can’t ask for more [from providers], since they are already too busy. E19*

## Discussion

This study maps and explores the integration of IPV within the Spanish decentralized health system in relation to the WHO recommendations on health sector responses to violence against women. It highlights the noteworthy progress achieved in a short period of time, especially in terms of legislation, high-level policy-making, and the development of a national coordination structure for learning, sharing and building consensus. Strategies to facilitate implementation of overarching policies at the level of health services have been put in place, especially in terms of the development of guidelines and efforts towards sensitizing and training health providers. National structures made strong efforts to incorporate regional initiatives, and reach consensus and strengthen cohesion, but the large differences between regions show that there is still work ahead. In light of the WHO recommendations, some challenges remain, such as the strengthening of monitoring systems, intersectorial coordination, and primary prevention actions.

Results show that RHSs align with the national level in terms of passing IPV legislation that includes the health sector. According to street-level bureaucrat theory, health policy implementation is highly dependent on individual civil servants; they play an important role and should be taken into account [41,42]. Getting these civil servants “on board” is paramount when potentially controversial programs are to be integrated. This study shows that in Spain, interventions geared towards IPV integration have considered the key role of these actors. The interventions have been participatory and in-line with a bottom-up approach to policy implementation [41,43]. Great efforts have been put into building a network of sensitized people with expertise on IPV who are convinced of the need to integrate it into the health system and motivated to take on this task.

Protocol or clinical guidelines were present in all regions. The aim of protocols was to guide the health providers’ work in terms of IPV, and they can also be seen as a marker of political commitment. However, participants in this study vacillated between considering protocols as the perfect tool to ensure an adequate response and considering that responding to IPV demanded much more creativity and competence from providers. This is in-line with other studies that indicate that dealing with and responding to IPV is an emotionally charged issue that involves a great deal of uncertainty [15,44]. Managing such uncertainty and adapting to the different needs of a diverse group of survivors of IPV can be supported by good guidelines, but it also requires investment in training and sensitizing providers.

Training has been a cornerstone for enabling providers to have sufficient skills to detect and manage IPV. However, available indicators do not facilitate evaluating the impact of those programs, because percentages of trained providers per region were not available, and few RHSs have published training manuals whose contents could be assessed. Training actions demonstrate a great deal of creativity, combining different approaches, duration and sites. One weakness noticed was of the limited supervision and support after training, a key component of successful incorporation of training into clinical practice [9]. Another is related to the weak integration of IPV training components within university training, an issue that has also been reported elsewhere [15,19].

This study also shows that comprehensive IPV legislation does not immediately translate into changes in the structure of the health systems; i.e. the inclusion of IPV as a component of regional health plans was not generalized, and health system’s strategies aimed at prioritizing certain health programs–such as “program contracts”-seldom included IPV within their targets. That IPV policy has difficulty in terms of follow-through has been pointed out elsewhere [45]. It might be due to the hegemony of the biomedical approach in the majority of health systems, which might not facilitate a comprehensive inclusion of complex issues such as IPV [14,44].

This study also demonstrates that institutionalized change [26] such as shifts in policy, protocols and organizational practices and structures still remain to be developed. Individual willingness and voluntarism remains a strong driver for actions, and it should be acknowledged that this motivation has generated changes all over the country. However, sustaining programs that rely on individual motivation becomes difficult if organizational structures do not change [13,27,46]. In terms of institutionalization of policies and programs, budget assignment is key. For this study it was not possible to get information regarding the budget allocated to IPV activities within the health system. This could be a sign of the fact that such programs face uncertain and poorly established funding channels within the health systems. The study also shows that participants considered that austerity measures have a negative impact on IPV programs, both in general and within the health system.

There were certain issues that the WHO recommendations considered as non-negotiable and that were less frequently included in the protocol. One such issue was the prohibition of contact with the partner. There is international consensus that not contacting the partner and always ensuring the privacy and confidentiality of the woman is necessary in order to protect her from further harm. The role of the health providers is to ensure that women exposed to IPV receive the best care with respect to her privacy and confidentiality [1,6]. However, due to the way Spanish primary health care practices are organized, avoiding all contact with the partner of a woman exposed to IPV is difficult. General practitioners in Spain are assigned entire families (husband, wife and children), and consequently when they detect a case of IPV they usually have been in contact with the woman-and her partner-for a long time. As a result, “not contacting the partner” is virtually impossible. Even if general practitioners can avoid addressing the issue of IPV with the partner, they must always deal with the fact that the perpetrator of the abuse is also a patient. This problem can be solved either by assigning the partner to another doctor-which could raise suspicion-or by continuing to provide care for the partner without mentioning the issue of IPV. It is an issue for which clear guidelines are absent. Hegarty et al. comment on this issue [47], and future WHO recommendations should take into consideration how to best advise providers dealing with such situations.

Another aspect that was not explicitly addressed in the majority of the protocols is that of ensuring that clinical records are kept in a confidential place. This could be due to the fact that clinical records in Spain are electronic and paper copies are not kept. However, even with electronic records, problems with privacy and confidentiality can occur. The need to monitor treatment of children is an important component of the health sector response to IPV, but few regional protocols provide explicit advice on this. It is important to note that the new national protocol more explicitly addresses these two issues, and it is likely that a revision of the regional protocols will take place accordingly [48].

Routine inquiry during pregnancy has proven to be an effective strategy [49-51], and it is included in the WHO recommendations. However, routine inquiry during pregnancy was institutionalized and/or included in the protocols in only 3 RHSs. The new national protocol, unlike the previous one, incorporates clinical inquiry of all women-pregnant or not-and explicitly addresses the special vulnerability of women to IPV during pregnancy [48]. Probably, this will lead to an increase in the application of clinical inquiry during pregnancy.

The NCGBV, the working groups and the Observatory have developed a set of common indicators to assess the situation of IPV and monitor trends. However, accountability and monitoring systems still face many challenges. Some limitations refer to the methods used to collect and analyze data; different diagnostic codes are used to label IPV cases (maltreatment, interpersonal violence, spousal abuse), and a diagnostic code that is both sensitive and specific has still not been incorporated into the electronic clinical records. This has also been reported in one previous study in Asturias (Spain) and elsewhere [16,52]. Data on existing indicators varied enormously from region to region, and was very low when compared to prevalence data from other studies [33,53] pointing out not only differences in regional functioning but also inaccuracies in reporting systems. Information collected was neither provided to the health care team producing it, nor used for monitoring purposes. An additional weakness in Spanish RHSs was the inexistence of mechanisms to collect information on women’s perceptions of the services. Several studies show that women’s perceptions can be very different from providers’ perceptions, and since many studies show that women’s experiences with services are not positive, there is need to further consider their perspective in order to improve the services offered [14,22,54-56].

It is essential to note that information regarding prevention/promotion activities was scarce. On the one hand, since the majority of factors associated with IPV belong to the social sphere, it is difficult to determine the health sector role and what lies beyond its responsibilities [57]. On the other hand, both public health and primary health care focus on prevention and promotion [58]. In recent decades, primary health care in Spain-and elsewhere-has become less prevention-oriented and more medicalised. Increased demand has reduced consultation hours, further hindering the work of professionals at this level [59]. This scenario reduces the likelihood that the health system will institutionalize preventive actions regarding IPV, and may leave such actions dependent on the voluntarism of select professionals and services. It is however, hopeful that the new protocol includes a section on the Integral Approach to Health, as the base for adequate integration of an IPV response [48].

The literature shows that few national IPV laws explicitly address the responsibilities of the health sector [28,29]. This paper constitutes a first attempt to establish concrete criteria to define and assess the responsiveness of health systems to IPV, beyond the statements contained in the legislation. By better defining such criteria it will be possible to monitor to what extent health services are responding to the needs of women exposed to IPV, and to establish mechanisms for improvement. We consider that these indicators could serve as a starting point to assess how health systems outside Spain are responding to IPV.

This paper highlights the issues in Spain that should be improved in order to better respond to the needs of women exposed to IPV. These issues include stronger institutionalization of the health care response to IPV such that the response offered to women is less dependent on professionals’ personal motivation, a better monitoring system that takes into account women’s perceptions, and a stronger focus on primary prevention. The differences between regional health systems also underscores the need to harmonize the strategies carried out, in order to ensure that the quality of health care received by a woman exposed to IPV is not dependent on the region where she lives.

### Limitations and strengths

This paper constitutes a first attempt to map and explore the integration of IPV within a health system. As with any first attempt, there are a number of limitations to be acknowledged, amongst them: 1) the selection of indicators is based on WHO recommendations and also on data available in Spain, and it is arguable whether they constitute the gold-standard for analyzing health system responsiveness to IPV, 2) information was collected at just one point in time, which does not enable a chronological picture of advances and regression, 3) it was not possible to collect data on some issues such as funding or actual practices that are very relevant for assessing a health system’s responsiveness to IPV, 4) in general, participants were highly motivated people, and as such they may have portrayed an overly positive picture of the Spanish health system’s response to IPV, and 5) the study portrays the Spanish health system response as the combination of the regional responses, without putting much focus on the national level aspects and the relationship between regions. Furthermore, it would have been interesting to explore why regional differences in health system’s response to IPV exist, but we did not collect that information during the interviews since the aim of the study was not to explore in depth regional differences, but to give an overview of the situation in Spain.

There are also a number of strengths to be highlighted: 1) the research design allowed for triangulation of information from the public document review and the qualitative interviews, which enriched the findings and allowed the description of both the broad (mapping) and deeper (exploring) perspectives, 2) the emergent design allowed data collection and analysis steps to be responsive to emergent findings, 3) the study collects disaggregated information at the regional level, allowing comparisons, and 4) the case selected (Spain) offered a rich scenario due to the number and diversity of strategies that have been implemented as part of the health care response to IPV.

## Conclusion

The study puts into evidence different levels of achievements between RHSs and also between the five areas assessed. Progress has been made at the level of policies, while it is less outstanding regarding health service delivery, and very limited in terms of preventive actions. There are still challenges remaining for a comprehensive integration of IPV response within the Spanish health system such as, 1) establishment of good coordination with other sectors despite their different approaches to IPV, 2) incorporation of new issues, such as routine inquiry in antenatal care, into the protocols, 3) strengthening mechanisms to improve accountability systems in order to enhance their specificity as well as the use of data for reorienting programs, 4) exploring ways to strengthen work on prevention and promotion activities, and 5) sustaining the progress made in the face of increased uncertainty and shrinking resources due to the recently implemented austerity measures.

## Competing interests

The authors declare that they have no competing interests.

## Authors’ contributions

IG participated in the design of the study, data collection and analysis, wrote the first draft of the manuscript, and was mainly responsible for incorporating co-authors suggestions and changes. CVC, AÖ and KE participated in the design of the study, and the interpretation of the data, critically reviewed drafts of the paper and approved its final version. FM and EBV participated in data collections, analysis and interpretation, critically reviewed drafts of the paper and approved its final version. All authors read and approved the final manuscript.

## Pre-publication history

The pre-publication history for this paper can be accessed here:

http://www.biomedcentral.com/1471-2458/13/1162/prepub

## Supplementary Material

Additional file 1 **List of main public documents reviewed.** Presents a list of the main documents reviewed for each of the 17 regional health systems. Click here for file

Additional file 2 RATS guidelines for qualitative research, applied to assess the manuscript Mapping and exploring health systems’ response to intimate partner violence in Spain. Click here for file
