# Supplementary material for: Mapping and exploring health systems’ response to intimate partner violence in Spain
Source: BMC Public Health. 2013 Dec 10;13:1162. doi: 10.1186/1471-2458-13-1162 (PMC3890595; doi:10.1186/1471-2458-13-1162)
Supplement: Additional file 1 — List of main public documents reviewed. Presents a list of the main documents reviewed for each of the 17 regional health systems. [file 1471-2458-13-1162-S1.pdf]

Additional file 1. List of main public documents reviewed.

REGION	AUTONOMIC LAW	PROTOCOLS	HEALTH PLANS
**Andalucía**	Ley 13/2007, de 26 de noviembre, de medidas de prevención y protección integral contra la violencia de género	Protocolo Andaluz para la actuación ante la violencia de género (2008)	3er Plan Andaluz de Salud 2003-2008
**Aragón**	Ley de la Comunidad Autónoma de Aragón 4/2007, de 22 de marzo, de Prevención y Protección Integral a las Mujeres Víctimas de Violencia	Protocolo para la detección y atención de la violencia de género en atención primaria (2007)	Plan Estratégico de Salud 2012-2015
**Asturias**	Ley del Principado de Asturias 2/2011, de 11 de marzo, para la igualdad de mujeres y hombres y la erradicación de la violencia de género	Protocolo sanitario para mejorar la atención a las mujeres víctimas de violencia de género (2007)	Plan de Salud para Asturias: La salud como horizonte 2004-2007
**Baleares**	Ley 12/2006, de 20 de septiembre para la mujer	Recomendaciones para la actuación sanitaria ante la violencia de género en la comunidad autónoma de las Ills Balears (2009)	Pla de Salut de les Illes Balears 2003/2007
**Canarias**	Ley16/2003 de prevención y protección integral de las mujeres contra la violencia de género. Artículo 10: Obligaciones de los centros y servicios sanitarios y de servicios sociales	Protocolo de actuación ante la violencia de género en el ámbito domestico (2003)	Plan de Salud 2004-2008: Más salud, mejores servicios
**Cantabria**	Ley de Cantabria 1/2004, de 1 de abril, integral para la prevención de la violencia contra las mujeres y la protección de sus víctimas	Protocolo de actuación sanitaria ante los malos tratos	Plan de Salud de Cantabria 1996-2000

	(Articulo 20-Atencion Sanitaria)		
**Castilla la Mancha**	Ley 5/2001 de 17-05-2001 de prevención de malos tratos y de protección de las mujeres maltratadas	Protocolo de actuación en atención primaria para mujeres víctimas de malos tratos (2005)	Plan de Salud de Castilla de la Mancha 2001-2010
**Castilla León**	Ley 13/2010 de 9 de diciembre contra la violencia de género en Castilla y León	Violencia contra las mujeres en la pareja. Guía de práctica clínica (2010)	III Plan de Salud Castilla y León (2008-2012)
**Catalunya**	LEY 5/2008, de 24 de abril, del derecho de las mujeres a erradicar la violencia machista.	Protocolo de actuación sanitaria ante los malos tratos (2005)<br><br>Protocolo para el abordaje de la violencia machista en el ámbito de salud en Cataluña (2009)	Plan de Salud de Cataluña 2011-2015
**C. Valenciana**	La ley Orgánica 1/2004 de 28 diciembre. Capitulo 1	Protocolo de actuación para la atención de las mujeres víctimas de violencia de género (2008)	Plan de Salud de la Comunitat Valenciana 2010/2013
**Extremadura**	Ley 8/2011, de 23 de marzo, de Igualdad entre Mujeres y Hombres y contra la Violencia de Género en Extremadura	Actuación Sanitaria ante la Violencia de Género-Protocolo de vigilancia epidemiológica de la violencia de género en Extremadura (2010)	Plan de Salud 2009-2012
**Galicia**	Ley de la Comunidad Autónoma de Galicia 11/2007 de 27 de julio, gallega para la prevención, y el tratamiento integral de la violencia de género	Guia técnica do proceso de atención as mulheres en situación de violencia de xenero (2009)	Plan de Salud de Galicia 2002-2005

**La Rioja**	Ley 3/2011, de 1 de marzo, de prevención, protección y coordinación institucional en materia de violencia en La Rioja	Protocolo de actuación sanitaria ante la violencia contra las mujeres (2010)	II Plan de Salud, La Rioja 2009-2013
**Madrid**	Ley 5/2005, de 20 de diciembre, Integral contra la Violencia de Género de la Comunidad de Madrid	Guía de apoyo en atención primaria para abordar la violencia de pareja hacia las mujeres. (2008)	Plan de promoción de la salud y prevención 2011-2013.
**Murcia**	Ley 7/2007, de 4 de abril, para la igualdad entre mujeres y hombres, y de protección contra la violencia de género en la Región de Murcia	Protocolo para la detección y atención de la violencia de género en atención primaria (2007)	Plan de Salud 2010-2015 de la Región de Murcia
**Navarra**	Ley Foral 22/2002 de 2 de julio, para la adopción de medidas integrales contra la violencia sexista. Artículo 14: Atención Sanitaria.	Protocolos de actuación sanitaria ante los malos tratos domésticos, físicos, psicológicos y/o agresiones sexuales	Plan de Salud de Navarra 2006-2012
**País Vasco**	Ley 4/2005 de 8 febrero, para la igualdad de mujeres y hombres. Cap. VII del título III-Violencia contra las mujeres	Protocolo sanitario ante malos tratos domésticos	Plan de Salud del País Vasco 2002-2010

Some general sites

- Ministerio de Sanidad, Servicios Sociales e Igualdad (Informes sobre violencia de género 2005 to 2010) `
  http://www.msc.es/organizacion/sns/planCalidadSNS/e02_t03_Comision.htm
- El portal de la Asociación Español de Neuropsiquiatría Profesionales de Salud Mental (Guías sobre violencia de género de la CC.AA)
  http://www.aen.es/index.php?option=com_docman&task=cat_view&gid=424

Some sites of the autonomous regions

- El portal sanitario de la Región de Murcia — http://www.murciasalud.es
- Conselleria de Bienestar Comunitat de Valencia — http://www.bsocial.gva.es
- Consejería de Sanidad y Servicios Sociales (Cantabria) — http://www.saludcantabria.org/
- El portal del Gobierno de Canarias — http://www.gobcan.es/
- El portal de planificación sanitaria y las políticas de salud (País Vasco) — http://www.osakidetza.euskadi.net
- Portal de Salud Castilla y León — http://www.saludcastillayleon.es
- El portal Navarra — http://www.navarra.es
- Instituto Asturiano de la mujer — http://institutoasturianodelamujer.com
- El portal de la comunidad de Madrid — http://www.madrid.org/cs/Satellite?pagename=PortalSalud/Page/PTSA_home
