# Supplementary material for: Mapping and exploring health systems’ response to intimate partner violence in Spain
Source: BMC Public Health. 2013 Dec 10;13:1162. doi: 10.1186/1471-2458-13-1162 (PMC3890595; doi:10.1186/1471-2458-13-1162)
Supplement: Additional file 2 — RATS guidelines for qualitative research, applied to assess the manuscript Mapping and exploring health systems’ response to intimate partner violence in Spain. [file 1471-2458-13-1162-S2.doc]

| **ASK THIS OF THE MANUSCRIPT**  RATS guidelines for qualitative research, applied to assess the manuscript Mapping and exploring health systems’ response to intimate partner violence in Spain | **THIS SHOULD BE INCLUDED IN THE MANUSCRIPT** | EXPLANATION OF HOW WE HAVE ADDRESSED EACH POINT IN THE MANUSCRIPT |
| --- | --- | --- |
| **R Relevance of study question** |  |  |
| Is the research question interesting?  Is the research question relevant to clinical practice, public health, or policy? | Research question explicitly stated  Research question justified and linked to the existing knowledge base (empirical research, theory, policy) | The research question is stated in the Background section (see page 5). The Background section (pages 4 to 6) draws on the literature to show: 1) the important role of the health system in preventing and responding to IPV, and 2) the scarce research that explores the health sector response to IPV from a health system’s perspective.  We have updated the reference for the global estimates of intimate partner violence against women. In the previous version we cited the WHO multi-country study conducted in 2000-2003. Now we cite the most recent WHO estimates on domestic violence, from June 2013, just after we submitted the article (WHO, 2013. Global and regional estimates of violence against women: prevalence and health effects of intimate partner violence and non-partner sexual violence. Geneva: WHO). |
| **A Appropriateness of qualitative method** |  |  |
| Is qualitative methodology the best approach for the study aims?   - *Interviews:* experience, perceptions, behaviour, practice, process - *Focus groups:* group dynamics, convenience, non-sensitive topics - *Ethnography:* culture, organizational behaviour, interaction - *Textual analysis:* documents, art, representations, conversations | Study design described and justified i.e., why was a particular method (e.g., interviews) chosen? | We justified the use of a systematic revision of public documents (to map the situation in Spain, against a set of indicators that assessed what a health system’s response to IPV should look like) and qualitative interviews with key informants (to explore more in depth aspects that could not be explored just through a review of existing documents).  We justified the use of methods to respond the research question in the Research methodology section (pages 7 to 10). |
| **T Transparency of procedures**  *Sampling* |  |  |
| Are the participants selected the most appropriate to provide access to the type of knowledge sought by the study?  Is the sampling strategy appropriate? | Criteria for selecting the study sample justified and explained   - *theoretical:* based on preconceived or emergent theory - *purposive:* diversity of opinion - *volunteer:* feasibility, hard-to-reach groups | Regarding the documentary review, we attach an additional file describing the types of documents reviewed to show that we reviewed an ample range of documents, both those easily available through the internet and others that were only available as hard copies. See on page 8 and in additional file “List of main public documents reviewed”.  Regarding the interview participants, we explain our process of theoretical sampling. Informants were selected because we considered that they could give rich information regarding our research question. We selected civil servants at the managerial level, and not politicians, because the former remain in their positions for a longer time period, and they have been more directly involved in implementing the health system’s response to IPV in their regions. We have included this information on page 9. |
| *Recruitment* |  |  |
| Was recruitment conducted using appropriate methods? | Details of how recruitment was conducted and by whom | This information is included in the text. See on page 9, where it states:  “First contacts were facilitated through the National Observatory of Women’s Health and subsequently by interviewees themselves, through snowball sampling”. |
| Is the sampling strategy appropriate? |  | We think that the sampling strategy was appropriate. We looked for key informants with experience in the implementation of IPV policies in the health systems, both at the national level and at the regional level, since we thought that those informants would provide important information to understand the process. In fact, the findings show that the information provided through the interviews allowed for depth in the information collected through the documentary review. |
| Could there be selection bias? | Details of who chose not to participate and why | We have incorporated a sentence on page 9, stating that “All of the approached informants agreed to participate”. This could have been due to the fact that those were generally highly motivated people. Although, they also were critical in pointing out the challenges and barriers. We have now included this issue as a potential limitation. See page 28, where it reads:  “4) in general, participants were highly motivated people, and as such they may have portrayed an overly positive picture of the Spanish health system’s response to IPV” |
| *Data collection* |  |  |
| Was collection of data systematic and comprehensive? | Method(s) outlined and examples given (e.g., interview questions) | Yes, and we have now elaborated more on how data was collected (both for the documentary review and the interviews) (Pages 8 to 10, additional file 1, Figure 2). We also provide a reference to a methodological note recently published that better describes the methodology of the study (Goicolea et al, 2013. Applying the WHO recommendations on health-sector response to violence against women to assess the Spanish health system. A mixed methods approach). |
| Are characteristics of the study group and setting clear? | Study group and setting clearly described | We describe the setting on pages 6 and 7. We have elaborated more on the structure of the Spanish network of health care services, as per suggestion of Reviewer 1. We have also included Figure 1 describing the structure for monitoring the implementation of the IPV Law in the Spanish health system (See Figure 1). |
| Why and when was data collection stopped, and is this reasonable? | End of data collection justified and described | In this case we collected information from each regional health system; we collected data from secondary sources and complemented with qualitative interviews. We then conducted member checking with some of the participants (See this described in Figure 2). We stopped collecting data when we considered that we had enough information to describe the situation in Spain - we did not aim for thorough comparisons between regions that would have needed more interviews per region. |
| *Role of researchers* |  |  |
| Is the researcher(s) appropriate? How might they bias (good and bad) the conduct of the study and results? | Do the researchers occupy dual roles (clinician and researcher)? Are the ethics of this discussed? Do the researcher(s) critically examine their own influence on the formulation of the research question, data collection, and interpretation? | Yes, as in any research study, the background of the researcher influences the selection of the research question, methodology, etc. However, we tried to bracket our pre-understandings during data collection. Having a set of indicators based on international guidelines helped in being less subjective when assessing the public documents available. For the interviews, we had an interview guide and tried to conduct the interview in such a way as to avoid judgmental attitudes or stating our values or opinions. |
| *Ethics* |  |  |
| Was informed consent sought and granted? | Informed consent process explicitly and clearly detailed | Yes, written informed consent was obtained from all participants prior to conducting the interviews (see on page 10) |
| Were participants’ anonymity and confidentiality ensured? | Anonymity and confidentiality discussed | Yes, this was discussed when the participant was asked for the written informed consent, and it was also stated in the consent form that was given to each participant (see on page 10). |
| Was approval from an appropriate ethics committee received? | Ethics approval cited | Yes, the study was presented and approved by the Bioethics committee of the University of Alicante (Spain). See on page 10. |
| **S Soundness of interpretive approach**  *Analysis* |  |  |
| Is the type of analysis appropriate for the type of study?   - *thematic:* exploratory, descriptive, hypothesis generating - *framework:* e.g., policy - *constant comparison/grounded theory:* theory generating, analytical   Are the interpretations clearly presented and adequately supported by the evidence? | Analytic approach described in depth and justified  *Indicators of quality:* Description of how themes were derived from the data (inductive or deductive)  Evidence of alternative explanations being sought  Analysis and presentation of negative or deviant cases | We chose qualitative content analysis, focusing on the manifest content because we aimed for description. We followed Graneheim and Lundman’s approach to qualitative content analysis. This is described on page 10. Since we already had five areas that we wanted to explore, qualitative content analysis allowed us to select the meaning units referring to each of those content areas and to proceed with the coding and searching for categories within each content area. This allowed us to combine the information gathered from the documentary review with the categories emerging from the qualitative content analysis. Since we aimed for description and not theory generation or the identification of discourse, we think that the method chosen was appropriate. |
| Are quotes used and are these appropriate and effective? | Description of the basis on which quotes were chosen  Semi-quantification when appropriate  Illumination of context and/or meaning, richly detailed | We chose the quotes in order to show that our description of the categories emerged from the data. There are a number of quotes from different participants that we think help show the reader the richness of the data while retaining the voices of the participants. |
| Was trustworthiness/reliability of the data and interpretations checked? | Method of reliability check described and justified e.g., was an audit trail, triangulation, or member checking employed? Did an independent analyst review data and contest themes? How were disagreements resolved? | Various researchers with different professional backgrounds and different levels of familiarity with the context were involved in the analysis, which enhanced trustworthiness. Member checking was conducted, and the use of both secondary data and interviews served to triangulate the information. This is described on page 10, and in Figure 2. This is also further described in the reference that is now included (Goicolea et al, 2013) |
| *Discussion and presentation* |  |  |
| Are findings sufficiently grounded in a theoretical or conceptual framework?  Is adequate account taken of previous knowledge and how the findings add? | Findings presented with reference to existing theoretical and empirical literature, and how they contribute | In the Discussion section we contrast our findings with existing literature. We provide references from different authors knowledgeable on health system’s response to IPV both in Spain and internationally, such as Feder G, Colombini M, Tower M, O`Campo P, Hegarty K, O’Reilly R. See pages 22 to 28. |
| Are the limitations thoughtfully considered? | Strengths and limitations explicitly described and discussed | Yes, we have a section on Limitations and strengths. See on pages 28, 29. |
| Is the manuscript well written and accessible? | Evidence of following guidelines (format, word count)  Detail of methods or additional quotes contained in appendix  Written for a health sciences audience | We have included the usual sections of Background, Methods, Results, Discussion, and Conclusion. We have adhered to the style and guidelines of BMC Public Health. |
| Are red flags present? These are common features of ill-conceived or poorly executed qualitative studies, are a cause for concern, and must be viewed critically. They might be fatal flaws, or they may result from lack of detail or clarity. | *Grounded theory:* not a simple content analysis but a complex, sociological, theory generating approach  *Jargon:* descriptions that are trite, pat or jargon filled should be viewed sceptically  *Over interpretation:* interpretation must be grounded in "accounts" and semi-quantified if possible or appropriate  *Seems anecdotal, self evident:* may be a superficial analysis, not rooted in conceptual framework or linked to previous knowledge, and lacking depth  *Consent process thinly discussed:* may not have met ethics requirements  *Doctor-researcher:* consider the ethical implications for patients and the bias in data collection and interpretation | We think that our study does not present any “red flags”. There are limitations that we acknowledge in the Limitations and strengths section. |
